# Dental Caries Status and Associated Factors Among Adults in Southeastern Iran: Findings From the Zahedan Adult Cohort Study

**DOI:** 10.1002/cre2.70236

**Published:** 2025-10-07

**Authors:** Somaye Ansari Moghadam, Razie Keikha Arya, Hassan Okati‐Aliabad, Mahdi Mohammadi, Alireza Ansari‐Moghaddam

**Affiliations:** ^1^ Department of Periodontology Oral and Dental Disease Research Center Zahedan University of Medical Sciences Zahedan Iran; ^2^ Oral and Dental Disease Research Center Zahedan University of Medical Sciences Zahedan Iran; ^3^ Health Promotion Research Center Zahedan University of Medical Sciences Zahedan Iran

**Keywords:** dental caries, DMFT index, oral health, risk factors

## Abstract

**Objectives:**

Dental caries remains a major public health concern globally. This study aimed to assess the dental caries status and its associated factors among adults in Southeastern Iran.

**Material and Methods:**

This cross‐sectional study included 10,016 adults aged 35–70 years who participated in the Zahedan Adult Cohort Study (ZACS). The status of dental caries was assessed using the decayed, missing, and filled teeth (DMFT) index. Data on sociodemographic characteristics, oral hygiene practices, presence of oral lesions, current cigarette smoking and drug use, and history of chronic condition were collected through valid questionnaires from the ZACS. One‐factor and multifactor general linear models were used to identify significant factors associated with DMFT.

**Results:**

In this study, 99.4% of participants had a DMFT score of 1 or higher, indicating a high lifetime experience of dental caries. The mean DMFT score was 17.3. Additionally, 47.91% of participants had filled teeth (1), and 78.7% had decayed teeth (DT), with the 35–40 age group showing the lowest mean DT score of 3.65. Conversely, individuals over 60 had the highest DMFT score of 22.88. In the multifactor model, DMFT significantly increased with age and decreased with higher education levels. DMFT also decreased from underweight to obese individuals. Furthermore, DMFT significantly decreased with increased daily tooth brushing, while cigarette smokers and drug users exhibited significantly higher DMFT scores.

**Conclusions:**

This study revealed significant variations in DMFT scores based on age, education, and oral hygiene practices among adults in Southeastern Iran, highlighting the importance of consistent dental brushing. The findings suggest the need for targeted public health interventions, particularly for smokers, drug users, and less educated populations, to address oral health challenges and reduce dental caries prevalence.

## 1 Introduction

Oral diseases, including dental caries, periodontal disease, tooth loss, and oral cancers, pose significant global health and economic burdens, particularly affecting marginalized groups and those in low‐income countries. Despite being preventable, these chronic conditions persist due to social inequalities, inadequate funding, and limited access to care, resulting in severe personal consequences and substantial economic strains on families and healthcare systems (Peres et al. 2019). Oral diseases can significantly impact individuals' well‐being and quality of life. Dental issues, causing pain and discomfort, disrupt essential functions such as chewing, talking, and smiling, consequently affecting social roles (Baiju et al. 2017). Demographic shifts, such as population growth and aging, have led to a substantial increase in the cumulative burden of oral conditions in recent years (Kassebaum et al. 2017).

In 2017, on a global scale, there were 3.5 billion oral conditions, including 2.3 billion untreated caries in permanent teeth, and 267 million individuals experiencing complete tooth loss (Bernabe et al. 2020). The most widespread global oral health issue is untreated caries in permanent teeth. There is a notable shift in the burden of untreated caries from children to adults (Kassebaum et al. 2015). Globally, in 2019, untreated dental caries in permanent teeth increased by 48% since 1990, with 3.09 billion new cases (Qin et al. 2022). In India, dental caries prevalence remains relatively constant from 5 to 12 years (49%), followed by a gradual increase from 15 years (60%) to 35–44 years (78%), reaching its peak at 65–74 years (84%) (Janakiram et al. 2018). In Iran, decayed, missing, and filled teeth (DMFT) values significantly rose among all age groups and both genders from 1990 to 2017. During this period, the national all‐ages mean DMFT increased by nearly 58%, with decayed teeth (DT) and missing teeth (MT) rising by 84.5% and 31.6%, respectively. Filled teeth (Sadeghian Tafti et al. 2023) increased approximately 2.6 times, with the highest DT, MT, and FT observed in the 25–29, 60+, and 35–39 age groups in 2017 (Shoaee et al. 2024).

Globally, oral diseases impose a substantial economic burden, with annual direct costs reaching approximately US$ 387 billion and indirect costs totaling US$ 323 billion. Specifically, permanent tooth caries incur nearly US$ 22 billion in indirect costs worldwide (Jain et al. 2023).

Numerous studies have explored the prevalence of dental caries and its associated risk factors. A study on Egyptian adults found that 86.63% had dental caries experience. Age, body mass index (BMI), socioeconomic status (SES), education level, and brushing frequency were significant risk factors associated with dental caries prevalence among this population (Abbass et al. 2019). In a population‐based study in Norway, the mean DMFT was 15.1. Regular tooth brushing twice daily, avoidance of sugar‐containing soft drinks, and annual dental service attendance were associated with a reduced likelihood of experiencing caries (Oscarson et al. 2017).

Caries experience in a Portuguese population was 91.1%, with a higher prevalence in females. Risk indicators for dental caries included age, occupation, overweight status, never visiting a dentist, and self‐reported weak teeth status (Guerreiro et al. 2023). The dental caries survey among Uruguayan adults and elders unveiled a mean DMFT of 15.20 and 24.12 for the 35–44 and 65–74 age groups, respectively. Disadvantaged backgrounds were linked to a heightened prevalence of dental caries, underscoring a concentrated burden within this population (Álvarez et al. 2015). Investigations into dental caries among the Saudi population reveal that adults aged 30–45 years have a maximum caries prevalence of 98% with a DMFT of 14.53, while older individuals exhibit the highest DMFT score of 24.3 (Al‐Ansari 2014).

Dental caries, now classified as a non‐communicable disease (NCD), share risk factors with other chronic conditions. Its prevention not only addresses oral health but also contributes to the control of broader NCDs (Twetman 2018; Pitts et al. 2021; Giacaman et al. 2022). A recent study revealed that individuals with at least one dental caries had a higher risk of stroke and death, though not for coronary heart disease (CHD), with more decayed, missing, and filled surfaces significantly associated with stroke and death (Sen et al. 2024).

The prevalence of dental caries varied significantly across different regions, age groups, and SESs (Borg‐Bartolo et al. 2022), necessitating comprehensive studies to understand the specific dynamics within diverse populations. This study explored dental caries status, along with its correlated factors, among adults in Southeastern Iran, highlighting the characteristics of oral health in this geographical context.

## 2 Methods

### 2.1 Study Design and Sample

This cross‐sectional study used baseline data from the Zahedan Adult Cohort Study (ZACS), conducted in southeastern Iran. The ZACS was established to investigate NCDs and their associated risk factors among adults aged 35–70 years living in Zahedan. A total of 10,016 men and women were enrolled between October 2015 and January 2019. The sample size for this study was based on the baseline population of the ZACS, which is a regional branch of the nationwide Prospective Epidemiological Research Studies in Iran (PERSIAN) cohort. The target sample size for each cohort center was approximately 10,000 adults aged 35–70 years to ensure adequate statistical power for a wide range of health‐related outcomes, including NCDs and their risk factors. This large, population‐based sample provides sufficient precision to estimate the prevalence of dental caries and to examine associations with various demographic, behavioral, and health‐related factors. Participants were recruited through a multistage stratified sampling method to ensure socioeconomic diversity. In the initial stage, city zones were identified in collaboration with healthcare experts and classified into low, middle, and high socioeconomic areas. Subsequently, three districts were randomly selected—one from each category—and all eligible residents within these zones were invited to participate.

Inclusion criteria included Iranian citizenship, age between 35 and 70 years, a minimum of 9 months' residence in Zahedan (or at least 1 year for migrants from other regions), willingness to complete baseline assessments, and availability for follow‐up. Individuals with severe physical or mental illnesses that precluded their ability to complete the questionnaires or attend the cohort center were excluded. Further details on the rationale, objectives, and design of the study have been previously published (Shahraki‐Sanavi et al. 2022). The ZACS was conducted in accordance with the ethical principles outlined in the Declaration of Helsinki. All participants provided written informed consent before enrollment. The ZACS protocol was reviewed and approved by the Ethics Committee of Zahedan University of Medical Sciences (Ethics Code: IR.ZAUMS.REC.1393–96451).

### 2.2 Measurements

The outcome variable in the study was the DMFT index, serving as a measure of dental caries. Trained individuals conducted the examination and recorded the oral and dental conditions of the participants. Participants were seated on a mobile chair and underwent examination using an oral mirror and probe in the presence of artificial light. To compute DMF, the following criteria were employed: Teeth with carious lesions or caries requiring restoration were designated as D. Removed teeth due to decay were tallied as M. Temporary or permanent tooth fillings, or incomplete fillings without decay, were recorded as F. Teeth repaired for reasons other than caries were not considered in this category. Teeth excluded from the DMF calculation encompassed hidden teeth, congenitally MT, extra teeth, and those remaining as deciduous teeth in the permanent dentition, all excluded for reasons other than caries.

A tooth that has undergone a root canal was classified as FT if it had a veneer, repair, or prosthesis. Otherwise, it was classified as DT. A healthy tooth shortened only for use as a bridge base was not counted as MT, as its modification was not due to caries. For individuals with dental implants, if the implant was due to decay, it was considered an MT. Teeth with caries or caries with restoration were counted as DT. Temporary or permanent fillings, or incomplete fillings that had not decayed, were counted as FT. Teeth restored for reasons other than caries were not counted as FT.

Data on sociodemographic characteristics (age, gender, education status, wealth score index), tooth brushing frequency (does not brush, sometimes, once daily, twice daily, three times daily), weekly frequency of flossing and mouthwash use, presence of oral lesions, current cigarette smoking and drug use (yes, no), and history of diabetes, hypertension, and menopause for women (yes, no) were collected through valid questionnaires from the ZACS. SES was determined through a principal components analysis (PCA) incorporating data on durable asset ownership, housing conditions, and education level. The resulting SES score, derived from the weighted sum of variables, categorized individuals into five quintiles, ranging from very poor to very rich.

The BMI of participants was calculated by dividing their weight in kilograms by the square of their height in meters (m²). Participants were classified into four categories based on BMI: obese (≥ 30 kg/m²), overweight (25–29.9 kg/m²), normal weight (18.5–24.9 kg/m²), and underweight (< 18.5 kg/m²).

### 2.3 Statistical Analysis

Quantitative variables were presented as mean ± SD (standard deviation), while qualitative variables were described as number (percentage). One‐factor and multifactor general linear models were employed to identify significant factors associated with DMFT. The analysis was performed using SPSS version 28, considering a *p* value less than 0.05 as statistically significant in all analyses.

## 3 Results

A total of 10,016 adults, with a mean age of 50.4 ± 9.2 years, participated in the study. Among them, 39.1% were female, 21.8% were illiterate, 19% were drug users, 5.6% were cigarette smokers, and 17.9% used noncigarette tobacco. Of the study participants, 66.3% were overweight or obese, 18.9% were diabetic, and 25.6% were hypertensive. Approximately half of the participants brushed their teeth at least once a day, while 83.6% did not floss, and 96.2% did not use mouthwash. Individuals over 60 were less likely to have a higher education. Overweight increased with age, but obesity was more prevalent among those aged 41–60 years. This age group also exhibited better SES. The prevalence of denture use and lack of tooth brushing increased with age, while tooth brushing and flossing decreased with age. Although tobacco use did not vary with age, drug use increased among older individuals (Table 1).

**Table 1:** Frequency distribution of demographic factors, tobacco use, and oral hygiene habits based on age.

	Age (year)				
	35–40	41–50	51–60	61–70	Total
	*N* (%)	*N* (%)	*N* (%)	*N* (%)	*N* (%)
Gender					
Male	705 (37.6)	1225 (39.1)	1374 (40.1)	613 (38.8)	3917 (39.1)
Female	1172 (62.4)	1908 (60.9)	2053 (59.9)	966 (61.2)	6099 (60.9)
Education					
Illiterate	134 (7.1)	452 (14.4)	926 (27.0)	669 (42.4)	2181 (21.8)
Primary school	484 (25.8)	957 (30.5)	916 (26.7)	299 (18.9)	2656 (26.5)
Secondary school	369 (19.7)	606 (19.3)	461 (13.5)	263 (16.7)	1699 (17.0)
High school	606 (32.3)	683 (21.8)	728 (21.2)	227 (14.4)	2244 (22.4)
Associate/BSc degree	241 (12.8)	368 (11.7)	364 (10.6)	114 (7.2)	1087 (10.9)
MSc/PhD	43 (2.3)	67 (2.1)	32 (0.9)	7 (0.4)	149 (1.5)
Marital status					
Single	100 (5.3)	50 (1.6)	23 (0.7)	1 (0.1)	174 (1.7)
Married	1672 (89.1)	2845 (90.8)	3028 (88.4)	1296 (82.1)	8841 (88.3)
Dead wife/widow	56 (3.0)	182 (5.8)	342 (10.0)	270 (17.1)	850 (8.5)
Divorce	49 (2.6)	55 (1.8)	34 (1.0)	11 (0.7)	149 (1.5)
BMI					
Underweight	117 (6.2)	122 (3.9)	103 (3.0)	54 (3.4)	396 (4.0)
Normal	629 (33.5)	888 (28.4)	940 (27.4)	522 (33.1)	2979 (29.8)
Overweight	663 (35.3)	1155 (36.9)	1342 (39.2)	661 (41.9)	3821 (38.2)
Obese	467 (24.9)	966 (30.9)	1041 (30.4)	341 (21.6)	2815 (28.1)
Wealth score					
Very poor (1st quintile)	394 (21.0)	597 (19.1)	648 (18.9)	400 (25.3)	2039 (20.4)
Poor (1st quintile–2nd quintile)	460 (24.5)	637 (20.3)	686 (20.0)	411 (26.0)	2194 (21.9)
Average (2nd quintile–3rd quintile)	419 (22.3)	555 (17.7)	606 (17.7)	273 (17.3)	1853 (18.5)
Good (3rd quintile–4th quintile)	387 (20.6)	854 (27.3%)	849 (24.8)	297 (18.8)	2387 (23.8)
Rich (4th quintile–5th quintile)	217 (11.6)	490 (15.6)	638 (18.6)	198 (12.5)	1543 (15.4)
Diabetes					
No	1785 (95.1)	2728 (87.1)	2529 (73.8)	1077 (68.2)	8119 (81.1)
Yes	92 (4.9)	405 (12.9)	898 (26.2)	502 (31.8)	1897 (18.9)
Hypertension					
No	1765 (94.0)	2608 (83.2)	2222 (64.8)	856 (54.2)	7451 (74.4)
Yes	112 (6.0)	525 (16.8)	1205 (35.2)	723 (45.8)	2565 (25.6)
Menopause^a^					
No	1272 (98.9)	1742 (86.0)	424 (20.6)	8 (1.1)	3446 (56.5)
Yes	14 (1.1)	283 (14.0)	1635 (79.4)	719 (98.9)	2651 (43.5)
Brushing					
Does not brush	275 (14.7)	585 (18.7)	745 (21.8)	442 (28.0)	2047 (20.5)
Sometime	479 (25.5)	722 (23.1)	698 (20.4)	249 (15.8)	2148 (21.5)
Once daily	748 (39.9)	1123 (35.9)	999 (29.2)	336 (21.3)	3206 (32.0)
Twice daily	273 (14.6)	456 (14.6)	404 (11.8)	106 (6.7)	1239 (12.4)
Three times a day	62 (3.3)	79 (2.5)	70 (2.0)	26 (1.6)	237 (2.4)
Has denture	39 (2.1)	162 (5.2)	508 (14.8)	419 (26.6)	1128 (11.3)
Oral lesion					
No	1870 (99.7)	3117 (99.7)	3407 (99.5)	1566 (99.2)	9960 (99.6)
Yes	6 (0.3)	10 (0.3)	17 (0.5)	12 (0.8)	45 (0.4)
Number of flossing (times per week)					
0	1416 (75.5)	2522 (80.7)	2964 (86.6)	1458 (92.4)	8360 (83.6)
1–7	391 (20.8)	520 (16.6)	377 (11.0)	93 (5.9)	1381 (13.8)
8–14	47 (2.5)	66 (2.1)	52 (1.5)	17 (1.1)	182 (1.8)
More than 14	22 (1.2)	19 (0.6)	31 (0.9)	10 (0.6)	82 (0.8)
Number of mouthwash (times per week)					
0	1810 (96.5)	2993 (95.7)	3294 (96.2)	1527 (96.8)	9624 (96.2)
1–3	53 (2.8)	110 (3.5)	107 (3.1)	36 (2.3)	306 (3.1)
More than 3	13 (0.7)	24 (0.8)	23 (0.7)	15 (1.0)	75 (0.7)
Current cigarette smoking					
No	1798 (95.8)	2943 (93.9)	3221 (94.0)	1488 (94.2)	9450 (94.4)
Yes (daily)	79 (4.2)	190 (6.1)	205 (6.0)	91 (5.8)	565 (5.6)
Noncigarette tobacco use					
No	1518 (80.9)	2507 (80.0)	2878 (84.0)	1316 (83.3)	8219 (82.1)
Yes	359 (19.1)	626 (20.0)	548 (16.0)	263 (16.7)	1796 (17.9)
Drug use					
No	1655 (88.2)	2603 (83.1)	2661 (77.7)	1193 (75.6)	8112 (81.0)
Yes	222 (11.8)	530 (16.9)	765 (22.3)	386 (24.4)	1903 (19.0)

^a^ Menopause was not considered in the multifactor model because male individuals were missed.

Apart from gender, the mean DMFT increased with age. This trend was also observed for MT, whereas filled teeth showed an inverse trend, and DT did not show a clear trend. Generally, females had more filled teeth in contrast to DT. The mean number of DT was 4.01, FT was 1.87, and MT was 11.39. Additionally, 78.7% of participants had DT, with the 35–40 age group having the lowest mean DT score of 3.65 and the lowest DT prevalence of 19.5%. In contrast, individuals over 60 had the highest mean DMFT score of 22.88 (Table 2).

**Table 2:** Mean of decayed, missing, and filled teeth categorized by gender and age.

		DT	MT	FT	DMFT
Gender	Age (year)	Mean (SD)	Mean (SD)	Mean (SD)	Mean (SD)
Male					
	35–40	4.15 (4.85)	6.23 (5.75)	1.80 (2.71)	12.18 (7.70)
	41–50	4.56 (4.89)	9.24 (7.77)	1.75 (2.58)	15.55 (8.71)
	51–60	4.21 (4.54)	13.93 (9.82)	1.50 (2.67)	19.63 (9.19)
	61–70	4.13 (4.89)	17.66 (10.25)	1.00 (2.17)	22.79 (8.85)
Female					
	35–40	3.42 (3.74)	6.24 (5.42)	2.39 (3.08)	12.05 (6.87)
	41–50	3.94 (4.13)	8.85 (6.75)	2.48 (3.25)	15.27 (7.47)
	51–60	3.88 (4.00)	13.19 (8.70)	1.95 (2.95)	19.02 (8.11)
	61–70	4.14 (4.79)	17.79 (9.88)	1.05 (2.41)	22.97 (8.28)

The mean DMFT was 17.3 ± 8.8, and 99.4% of participants exhibited dental caries (DMFT ≥ 1). There was a significant increasing trend in DMFT with age (*p* < 0.001) and a decreasing trend with education (*p* < 0.001), BMI (*p* < 0.001), wealth (*p* < 0.001), brushing (*p* < 0.001), and number of flossing episodes per day (*p* < 0.001). The mean DMFT was significantly higher in males (*p* < 0.001), diabetic individuals (*p* < 0.001), hypertensive individuals (*p* < 0.001), cigarette smokers (*p* < 0.001), noncigarette tobacco users (*p* < 0.001), and drug users (*p* < 0.001) (Table 3).

In the multifactor model, a significant increasing trend of DMFT was observed with age (*p* < 0.001). Conversely, there was a decreasing trend in DMFT based on education level, with the mean DMFT significantly higher in illiterate (*p* < 0.001), primary (*p* = 0.030), and secondary (*p* = 0.037) school individuals compared with university graduates. A declining trend in DMFT was noted from underweight to obese individuals, with a significant difference between underweight (*p* < 0.001), normal (*p* < 0.001), and overweight (*p* = 0.026) compared with obese individuals. Additionally, DMFT significantly decreased with the number of toothbrushes used per day compared with no brushing (*p* < 0.001). Cigarette smokers (*p* < 0.001) and drug users (*p* < 0.001) exhibited significantly higher DMFT scores (Table 3).

**Table 3:** Factors associated with DMFT score.

Variables	DMFT	Mean comparisons		Multifactor model	
	Mean (SD)	*p* value	*p* value for trend	B ± SE	*p* value
Age (years)					
35–40	12.09 (7.14)	< 0.001	< 0.001	−6.41 ± 0.26	< 0.001
41–50	15.37 (7.93)			−3.97 ± 0.24	< 0.001
51–60	19.26 (8.56)			−1.74 ± 0.24	< 0.001
60–70	22.87 (8.59)			Reference	
Gender					
Male	18.04 (9.46)	< 0.001			
Female	16.77 (8.40)				
Education					
Illiterate	20.32 (8.84)	< 0.001	< 0.001	2.32 ± 0.60	< 0.001
Primary school	17.14 (8.72)			1.27 ± 0.58	0.030
Secondary school	17.21 (8.91)			1.24 ± 0.59	0.037
High school	16.24 (8.67)			1.07 ± 0.59	0.069
Associate degree or BSc	14.33 (7.90)			0.14 ± 0.60	0.813
MSc or PhD	12.83 (6.90)			Reference	
BMI					
Underweight	20.60 (9.72)	< 0.001	< 0.001	1.90 ± 0.41	< 0.001
Normal	18.49 (9.24)			1.20 ± 0.19	< 0.001
Overweight	16.89 (8.67)			0.40 ± 0.18	0.026
Obese	16.02 (8.26)			Reference	
Wealth score index					
Very poor	18.72 (9.20)	< 0.001	< 0.001		
Poor	17.66 (9.03)				
Average	16.99 (8.90)				
Rich	16.71 (8.53)				
Very rich	16.01 (8.27)				
Diabetes					
No	16.88 (8.82)	< 0.001			
Yes	18.94 (8.81)				
Hypertension					
No	16.63 (8.80)	< 0.001			
Yes	19.13 (8.74)				
Menopause^a^					
No	14.28 (7.55)	< 0.001			
Yes	20.01 (8.35)				
Brushing					
Does not brush	20.89 (8.86)	< 0.001	< 0.001	Reference	
Sometimes	14.69 (7.12)			−4.59 ± 0.21	< 0.001
Once daily	13.78 (6.66)			−4.86 ± 0.20	< 0.001
Twice daily	13.42 (6.25)			−5.05 ± 0.26	< 0.001
Three times a day	13.37 (7.05)			−5.08 ± 0.47	< 0.001
Oral lesion					
No	17.27 (8.86)	0.842			
Yes	17.53 (7.28)				
Number of flossing (times per week)					
0	18.12 (9.03)	< 0.001	< 0.001		
1–7	12.87 (6.32)				
8–14	13.30 (6.17)				
> 14	13.67 (6.50)				
Number of mouthwash (times per week)					
0	17.28 (8.88)	0.717	0.684		
1–3	16.92 (8.21)				
> 3	16.87 (8.71)				
Current cigarette smoking					
No	16.90 (8.71)	< 0.001		−2.98 ± 0.35	< 0.001
Yes (daily)	23.36 (8.98)			Reference	
Noncigarette tobacco use					
No	17.05 (8.79)	< 0.001			
Yes	18.29 (9.05)				
Drugs use					
No	16.01 (8.31)	< 0.001		−3.12 ± 0.21	< 0.001
Yes	22.62 (9.10)			Reference	
Alcohol consumption					
No	17.20 (8.82)	< 0.001			
Yes	19.99 (9.86)				

Abbreviations: B, coefficient of the regression model; SE, standard error of the coefficient.

a Menopause was not considered in the multifactor model because male individuals were missed.

## 4 Discussion

In this study, 99.4% of participants had experienced dental caries (DMFT ≥ 1), indicating an extremely high lifetime experience of dental caries in the population. This finding highlights a significant public health concern, suggesting widespread exposure to caries risk factors or insufficient access to preventive oral healthcare. The high prevalence underscores the urgent need for targeted oral health interventions, including education on proper hygiene practices, improved access to dental services, and policies promoting early detection and prevention. The mean DMFT of 17.3 among adults in Southeastern Iran indicates a significant burden of dental caries in this population, highlighting substantial oral health challenges. This finding is consistent with a multicenter study involving approximately 129,000 individuals over the age of 35 from 14 different provinces in Iran, which reported an average DMFT index of 18 (Najafi et al. 2020). In the Chilean adult population, the average DMFT was 15.06 for the 35–44 age group and 21.57 for the 65–74 age group (Urzua et al. 2012). This result is almost similar to the findings of our study. A study of the Norwegian adult population, conducted with 4913 participants aged 19 years and older, reported an average DMFT index of 14.9 (Rødseth et al. 2023). The difference in the mean DMFT index between the Zahedan and Norwegian populations can be partly attributed to variations in the age range of participants. Additionally, factors such as access to preventive dental services, frequency of dental visits, oral health literacy, dietary habits, and the prioritization of oral health within the public healthcare system may play significant roles.

In this study, age‐related variations in DMFT were notable. The observed increasing trend in DMFT with age underscores the significant impact of aging on oral health. Higher DMFT scores in individuals over 60 years emphasize the cumulative effects of aging on oral health. Previous studies have shown similar trends, with higher DMFT scores in older age groups (Najafi et al. 2020; Abdel Fattah et al. 2022). This finding aligns with the well‐established understanding that dental caries tend to accumulate over a person's lifespan. Accumulated periodontal destruction increases the risk surfaces for caries, and the consequences of treatments further heighten susceptibility to caries development (López et al. 2017). Additionally, evidence suggests that dental hygiene behaviors decrease with age (Jahangiry et al. 2020).

In the current study, the decreasing trend in DMFT with higher education levels is a crucial finding, indicating that individuals with lower educational attainment, such as those who are illiterate or have only primary or secondary school degrees, experience a higher burden of dental caries. In line with this finding, a previous study among adults in Ethiopia found that education level was negatively associated with dental caries experience (Bogale et al. 2021). Evidence suggests a correlation between educational level and oral health knowledge (Márquez‐Arrico et al. 2019), which may explain this association to some extent. Individuals with lower education levels likely face challenges in accessing oral health information, adopting preventive practices, and seeking timely dental care, contributing to the observed trends in DMFT.

This study revealed that individuals with higher SES exhibited lower DMFT scores in the one‐factor model. Globally, sociodemographic inequality in 2019 was attributed to 64.6 million prevalent cases of caries in permanent teeth (Wen et al. 2022). Additionally, a comprehensive cohort study in Iran found a higher prevalence of dental caries among socioeconomically disadvantaged adults (Najafi et al. 2020). A similar pattern of notable socioeconomic inequalities in dental caries was observed among Chinese adults (Chang et al. 2023).

In the current study, an inverse relationship was observed between BMI and DMFT score. This inverse relationship indicates that individuals with higher body weight (overweight and obese) may experience lower DMFT scores compared with their underweight or normal‐weight counterparts. In the current study, we observed that as BMI increased, participants tended to exhibit more favorable brushing behavior and reported lower rates of cigarette and drug use, both of which are known contributors to poor oral health. These healthier behaviors may partially explain the lower DMFT scores among overweight and obese individuals compared with those with lower BMI. A study on Korean adults found an inverse association between overweight or obesity and the prevalence or severity of dental caries. Furthermore, the caries index showed an inverse relationship with increasing body fat quartiles, as subjects with higher waist circumference were less prone to experiencing tooth decay (Song et al. 2017).

In the present study, dental brushing, flossing, and mouthwash use were associated with DMFT scores in a one‐factor model, but only brushing was associated with DMFT scores in a multifactor model. The significant decrease in DMFT with an increase in the number of daily brushes highlights the crucial role of consistent dental brushing practices in preventing dental caries. Evidence shows that poor tooth brushing habits are an independent risk factor for dental caries (Teshome et al. 2021), while individuals who brush their teeth more frequently tend to experience fewer dental caries (Kumar et al. 2016).

In the present study, the significantly higher DMFT scores among cigarette smokers and noncigarette tobacco users highlight the detrimental impact of these lifestyle factors on oral health. A systematic review and meta‐analysis revealed a correlation between tobacco smoking and an elevated risk of dental caries (Jiang et al. 2019). In alignment with this study, the Bandare‐Kong Cohort Study unveiled a higher DMFT index in individuals who smoked cigarettes, hookahs, or both, compared with nonsmokers (Rafati et al. 2023). Smoking promotes cariogenic microorganism growth by affecting saliva, reducing its buffer capability, and altering chemical and bacterial components. This, in turn, creates an environment conducive to the development of dental caries (Wu et al. 2019). According to recent national data from the STEPS surveys, the prevalence of current tobacco smoking among Iranian adults is approximately 14%, with higher rates in certain subpopulations and geographic regions (Sohrabi et al. 2020). Iran has made significant progress in tobacco control within the Eastern Mediterranean Region through comprehensive policy implementation, ranking among the leading countries in this field. The country has achieved the highest score for smoke‐free public places, allocated the highest tobacco control budget relative to GDP in the region, fully enforced bans on tobacco advertising, and introduced large health warning labels on tobacco products. Additionally, Iran is recognized as one of the top countries in providing tobacco cessation support services.

Despite these achievements, there remains a gap in pricing policies. The average cost of a pack of cigarettes in Iran is still relatively low, which undermines efforts to reduce tobacco consumption. Increasing tobacco taxes and retail prices is a well‐established strategy in global tobacco control, and addressing this policy area could further strengthen Iran's efforts to curb smoking and its related health burdens (Saeedi et al. 2023).

The observed significantly higher DMFT scores among drug users underscore the potential oral health implications associated with drug use. In this study, the majority of drug‐using participants (98.5%) reported opium use, reflecting its widespread prevalence in the region. Opium use may adversely affect oral health through several mechanisms. It is commonly associated with xerostomia (dry mouth), which reduces the protective effects of saliva and increases vulnerability to dental caries. Additionally, individuals who use opium often exhibit poor oral hygiene practices, increased cravings for sugary foods, and irregular dental attendance, all of which contribute to higher DMFT scores (Rossow 2021). Systematic reviews and meta‐analyses consistently indicate that individuals using drugs face caries risk approximately four times higher than nonusers (Yazdanian et al. 2020; Paisi et al. 2021). The impact may be attributed to various factors, including compromised oral hygiene practices (Shekarchizadeh et al. 2013) or reduced access to dental care (Baghaie et al. 2017) among substance abusers.

### 4.1 Practical Implications

The findings of this study underscore the need for practical and targeted public health interventions to address modifiable risk factors associated with dental caries in Southeastern Iran. Oral hygiene behaviors, particularly regular tooth brushing, were significantly associated with DMFT scores, suggesting that educational programs promoting proper oral care practices should be prioritized. These efforts should especially target older adults and individuals with lower educational attainment, who were found to be at higher risk. Furthermore, the association of higher DMFT scores with cigarette smoking and drug use highlights the importance of integrating oral health education into broader tobacco and substance use prevention initiatives. Expanding access to preventive dental services, particularly in underserved and socioeconomically disadvantaged regions such as Zahedan, is also essential. These findings call for comprehensive, community‐based interventions tailored to the specific cultural and social context of the population to effectively reduce the burden of dental caries and improve overall oral health outcomes.

### 4.2 Strengths and Limitations

This study has several strengths and limitations that should be acknowledged. Among its strengths, the large and representative sample drawn from the ZACS enhances the generalizability of the findings to the adult population of Southeastern Iran. The use of standardized protocols and trained dental examiners for clinical assessment improved the reliability of oral health measurements. In addition, the study examined a wide range of sociodemographic, behavioral, and health‐related variables, allowing for a comprehensive analysis of factors associated with dental caries.

However, some limitations must be considered. The cross‐sectional design precludes the establishment of causal relationships. Self‐reported data on behaviors such as oral hygiene practices, smoking, and drug use are subject to recall and social desirability bias. To minimize these effects, data were collected using validated questionnaires administered by trained interviewers under confidential conditions. While self‐reported oral hygiene practices are commonly used in large‐scale studies and offer practical value, they may not always reflect actual behavior. Alternative approaches, such as clinical assessment of plaque levels or biochemical verification of tobacco and drug use, could enhance accuracy but were not feasible in the context of this cohort. Future studies should consider incorporating objective measures to validate self‐reported behaviors and improve the reliability of findings. It is also important to note that data on participants' access to preventive dental care and the frequency of dental check‐ups, brushing technique, toothpaste type (fluoride exposure), sugar intake, and psychosocial factors (e.g., stress and dental anxiety) were not available in this study. These factors could significantly influence DMFT scores, and their absence represents a limitation. Future research should incorporate such variables to provide a more comprehensive understanding of dental caries risk and oral health disparities. Despite these limitations, the study provides important baseline data and highlights key areas for future research and public health intervention.

## 5 Conclusion

This study highlights significant variations in DMFT scores based on age, education level, and oral hygiene practices among adults in Southeastern Iran. The findings underscore the critical role of consistent dental brushing in reducing dental caries. Additionally, higher DMFT scores in smokers and drug users suggest the need for targeted public health interventions. The substantial differences in DMFT across various age groups emphasize the necessity for age‐specific dental health strategies. Comprehensive efforts are required to address the oral health challenges identified, particularly among less educated and underweight populations. Overall, improving oral health education and promoting better oral hygiene practices are essential to reducing the prevalence of dental caries in this region.

## Author Contributions

**Somaye Ansari Moghadam:** conceptualization, methodology, supervision, critical review of the manuscript. **Razie Keikha Arya:** writing – original draft preparation, data curation, revision. **Hassan Okati‐Aliabad:** conceptualization, writing – original draft preparation, study design, project administration. **Mahdi Mohammadi:** statistical analysis, data visualization, interpretation of results. **Alireza Ansari‐Moghaddam:** formal analysis, validation; writing – review and editing, supervision. All authors critically reviewed the manuscript for intellectual content and approved the final version.

## Ethics Statement

The current study received approval from the Ethics Committee of Zahedan University of Medical Sciences (Approval Code: IR.ZAUMS.REC.1401.399).

## Consent

All participants of the ZACS provided written informed consent.

## Conflicts of Interest

The authors declare no conflicts of interest.

## Data Availability

The data that support the findings of this study are available from the corresponding author upon reasonable request.
